# Impact of cardiac arrhythmia on velocity quantification by ECG gated phase contrast MRI

**DOI:** 10.1186/1532-429X-17-S1-Q16

**Published:** 2015-02-03

**Authors:** Michael Markl, Daniel C Lee, Jason Ng, James C Carr, Jeffrey J Goldberger

**Affiliations:** 1 Northwestern University, Chicago, IL, USA

## Background

Blood flow quantification using ECG gated phase contrast (PC) MRI has proven to be a useful clinical tool for the evaluation of cardiovascular pathologies such aortic valve disease or cardiac shunts^1^. However, data are acquired in an ECG synchronized manner over multiple heart beats and the impact of cardiac arrhythmia, which can frequently be encountered in patients with atrial fibrillation (AF), on flow parameters is unclear. To assess the impact of arrhythmia and thus RR interval variability on ECG-gated PC MRI acquisitions we have performed a simulation study to systematically investigate the impact of beat-to-beat variations on ECG gated multi-beat flow imaging with MRI using real time in-vivo TEE data in 5 AF patients with known arrhythmia.

## Methods

Real-time 2-dimensional transesophageal echocardiography (TEE) in color flow Doppler mode was performed in five patients in AF (4 male, age=64±8.7 years). TEE data provided real-time left atrial (LA) and left ventricular (LV) flow velocities in 2-4 consecutive cardiac cycles with different RR-interval durations. PC-MRI acquisitions were simulated using k-space data, simulated from the TEE velocity measures, to construct time-resolved PC-MRI k-space data for a segmented sampling scheme typically used for ECG gated 2D PC MRI. Each simulation was repeated 100 times to minimize effects from data that may be weighted to a particular beat in the center of k-space. The resulting LA and LV velocities were compared to the average TEE velocities and to the TEE velocity data from individual cardiac cycles. Average velocity measurements were quantitatively compared using regions of interest (ROIs) in the LA and LV.

## Results

The average R-R interval duration for all five patients was 643 ± 161ms, with a range of 364-1000ms. Results of the simulation study (see figure) demonstrated that ECG gated flow MRI in patients with cardiac arrhythmia can reliably assess patterns of blood flow velocities that are consistently present over multiple heart beats (persistent or average flow patterns). ECG gated flow imaging with MRI could reproduce persistent average LA and LV mean velocities within 7.0-7.4% compared to TEE. Average TEE and simulated MR velocity measurements within the ROIs were not significantly different.

## Conclusions

PC-MRI velocity measurements in patients with varying R-R interval durations are not significantly different from time-averaged real-time velocity data for a typical segmented k-space data acquisition schemes. Though beat-to-beat variations in atrial velocities that were observed with TEE cannot be detected with ECG gated multi-beat PC MRI, it can reliably assess average flow patterns across multiple beats.

## Funding

AHA (12GRNT12080032) and NIH-NHLBI (1R21HL113895).
